# Risk Factors and Complications of Transfusion Following Partial Nephrectomy

**DOI:** 10.7759/cureus.112060

**Published:** 2026-07-04

**Authors:** Devki Patel, Vaishnavi J Patel, Mark E Quiring, Kedar Patel, Kimberly C Toumazos, Young Son, Thomas J Mueller

**Affiliations:** 1 Office of Research and Innovation, School of Medicine, Texas Tech University Health Sciences Center, Lubbock, USA; 2 Department of Family Medicine, Dell Medical School, The University of Texas at Austin, Austin, USA; 3 Department of Urology, University of North Texas Health Science Center, Fort Worth, USA; 4 Office of Undergraduate Research, The University of Texas at Austin, Austin, USA; 5 Department of Urology, University of Kentucky College of Medicine, Lexington, USA; 6 Department of Urology, Jefferson Health New Jersey, Stratford, USA

**Keywords:** blood transfusion, partial nephrectomy, perioperative risk factors, renal tumors, surgical complications

## Abstract

Background and objectives

Partial nephrectomy (PN) is the gold standard for small renal tumors, preserving nephrons without compromising oncologic outcomes. However, PN carries a higher complication rate, with postoperative bleeding being a notable risk. Despite its significance, data on transfusion risk factors remain limited. This study identifies variables associated with perioperative transfusion in patients undergoing PN.

Methods

A retrospective cohort study was conducted using the 2019-2020 American College of Surgeons (ACS) National Surgical Quality Improvement Program (NSQIP) database. Patients undergoing PN were identified and stratified by transfusion status. Preoperative, perioperative, and postoperative variables were analyzed using univariate and multivariate logistic regression models.

Results

Among 892 PN patients, 116 (13.0%) required transfusion. Transfused patients were older (p=0.026) and had higher rates of disseminated cancer (p=0.020), weight loss (p=0.036), bleeding disorders (p=0.025), and prior pelvic surgeries (p=0.014). Perioperative factors included longer operative times (p<0.001), concomitant procedures (p<0.001), and unplanned reoperations (p<0.001). Multivariate analysis identified concomitant procedures (OR: 2.27, p<0.001), prior chemotherapy (OR: 4.89, p=0.001), and unplanned reoperation (OR: 6.16, p<0.001) as significant predictors. Transfusion increased the odds of composite complications (OR: 1.20, p=0.041).

Conclusions

Preoperative and perioperative factors influence transfusion risk, which in turn increases complication rates. Careful preoperative assessment and surgical planning can minimize transfusion-related morbidity. Restrictive transfusion strategies and optimized patient selection may improve outcomes.

## Introduction

Partial nephrectomy (PN) has emerged as the gold standard treatment for small renal tumors, including renal cell carcinoma (RCC) [1]. Historically, radical nephrectomy (RN) was the standard surgical approach for renal tumors; however, the advent of imaging techniques and early cancer detection has led to an increased emphasis on maintaining renal function through PN. One advantage of PN is that it preserves nephrons and renal parenchyma without compromising oncologic outcomes [2]. Studies have shown that, for renal masses suspicious for RCC, survival rates for patients treated with PN were 75%-99%, compared to 71%-81% for those who underwent radical nephrectomy [3].

While PN offers advantages over RN in preserving renal function, it is not without complications, with PN having higher complication rates overall compared to RN [4]. One such complication is postoperative bleeding. Postoperative bleeding has been reported to occur in up to 5.25% of PN cases, versus 3%-10% of radical nephrectomy cases [5]. These postoperative bleeds often necessitate blood transfusions to manage blood loss [6]. Understanding the factors contributing to bleeding risk is crucial for both surgical planning and postoperative management. The current literature on the risk of blood transfusion during and after PN is limited. The primary objective of this study is to determine the risk of transfusion after PN and to identify variables associated with this risk. By identifying the factors associated with transfusion, we aim to enhance clinical decision-making and limit the occurrence of such complications.

## Materials and methods

Source of data

The American College of Surgeons (ACS) National Surgical Quality Improvement Program (NSQIP) is a database that adheres to the regulations set forth by the Health Insurance Portability and Accountability Act (HIPAA). The program aims to evaluate the quality of care provided after surgical procedures. It contains surgical data from approximately 702 hospitals. The NSQIP 2019-2020 database includes 1979379 cases. Of these, 892 patients who underwent PN were included in the analysis. Principal operative procedure cases were identified based on Current Procedural Terminology (CPT) codes. As the ACS NSQIP database is de-identified, this retrospective study was exempt from institutional review board approval.

Population of study and analysis

The NSQIP database was reviewed for patients who had undergone a PN in 2019 or 2020. Inclusion criteria included procedures with primary CPT codes of “NEPHRECTOMY PARTIAL” (CPT 50240) or “LAPAROSCOPY SURG PARTIAL NEPHRECTOMY” (CPT 50543). A total of 484 cases were performed in 2019 and 406 cases in 2020. A total of 1978487 patients did not meet the inclusion criteria and were therefore excluded from the study. The cohort was then subdivided based on whether patients received a blood transfusion perioperatively or postoperatively.

Analysis was performed comparing the transfusion and no-transfusion groups. Preoperative, perioperative, and postoperative variables were compared between the two groups. Minor complications included urinary tract infections, superficial surgical site infections, and transfusion rates. Major complications included unplanned reoperation, unplanned readmission, hospitalization longer than 30 days, deep surgical site infection, organ space surgical site infection, pneumonia, unplanned intubation, pulmonary embolism, mechanical ventilation for longer than 48 hours, progressive renal insufficiency, acute renal failure, stroke or cerebrovascular accident (CVA) with neurological deficit, cardiac arrest requiring cardiopulmonary resuscitation (CPR), myocardial infarction, deep vein thrombosis (DVT) or thrombosis requiring therapy, sepsis, septic shock, wound disruption, and *Clostridioides difficile* colitis. These categories were derived from a previous research study and clinical expertise [7]. For categorical variables, a chi-square analysis was used. An independent-samples t-test was used for continuous variables. To identify variables for the logistic regression model, we used the Random Forest algorithm to determine the importance of independent variables. Univariate and multivariate logistic regression analyses were then performed using these variables. Multivariate analysis was performed with perioperative and postoperative complications as dependent variables, using the non-transfused group as the referent. Statistical analyses were conducted using R version 4.0.2 (R Foundation for Statistical Computing, Vienna, Austria), and statistical significance was accepted at p<0.05.

## Results

Among the total cohort of 892 PN patients, 116 (13.0%) required a blood transfusion. Patients requiring blood transfusions were older on average (p=0.026), but there were no differences in race (Table 1). A history of disseminated cancer (p=0.020), greater than 10% body weight loss in the previous six months (p=0.036), bleeding disorders (p=0.025), and prior pelvic operations (p=0.014) were all associated with receiving a blood transfusion after PN (Table 2). Preoperative serum creatinine (p<0.001) and hematocrit (p<0.001) also varied significantly between the two groups (Table 3).

**Table 1:** Patient demographics and preoperative characteristics Significance was defined as p<0.05, and p-values were obtained using chi-square and t-test analyses. BMI: body mass index

	No transfusion, n=776	Transfusion, n=116	Total cohort, n=892	P-value	t-value or χ² value
Female	283 (36.5%)	51 (44.0%)	334 (37.4%)	0.146	2.12
Race					
White	418 (53.9%)	72 (62.1%)	490 (54.9%)	0.12	2.42
American Indian or Alaska Native participants	29 (3.7%)	2 (1.7%)	31 (3.5%)	0.405	0.71
Black or African American participants	68 (8.8%)	16 (13.8%)	84 (9.4%)	0.119	2.43
Native Hawaiian or Pacific Islander participants	0 (0%)	1 (0.9%)	1 (0.1%)	0.271	1.43
Asian	9 (1.2%)	1 (0.9%)	10 (1.1%)	1	0.02
Unknown/not reported	252 (32.5%)	24 (20.7%)	276 (30.9%)	0.014	6.06
Hispanic	49 (6.3%)	5 (4.3%)	54 (6.1%)	0.525	0.4
Inpatient	767 (98.8%)	116 (100%)	883 (99.0%)	0.504	1.58
Age of patient	60.171 (11.982)	62.845 (12.592)	60.519 (12.090)	0.026	-2.23
Height in inches	66.813 (4.0419)	66.172 (3.9038)	66.730 (4.0279)	0.11	1.62
Weight in lbs	203.80 (46.801)	199.10 (53.139)	203.19 (47.667)	0.323	0.95
BMI	32.065 (6.8111)	31.825 (7.7161)	32.034 (6.9312)	0.728	0.35

**Table 2:** Medical history and comorbid conditions Significance was defined as p<0.05; p-values were obtained using chi-square and t-test analyses. COPD: chronic obstructive pulmonary disease; SIRS: systemic inflammatory response syndrome

	No transfusion, n=776	Transfusion, n=116	Total cohort, n=892	P-value	χ² value
Diabetes mellitus					
Insulin dependent	70 (9.0%)	16 (13.8%)	86 (9.6%)	0.145	2.12
Non-insulin dependent	148 (19.1%)	19 (16.4%)	167 (18.7%)	0.571	0.32
Current smoker	141 (18.2%)	15 (12.9%)	156 (17.5%)	0.21	1.57
Dyspnea present	43 (5.5%)	12 (10.3%)	55 (6.2%)	0.072	3.23
Independent living status	771 (99.4%)	115 (99.1%)	886 (99.3%)	1	0.01
History of severe COPD	33 (4.3%)	5 (4.3%)	38 (4.3%)	1	<0.01
Congestive heart failure in 30 days before surgery	4 (0.5%)	2 (1.7%)	6 (0.7%)	0.381	0.77
Hypertension requiring medication	490 (63.1%)	83 (71.6%)	573 (64.2%)	0.097	2.76
Currently on dialysis (pre-op)	2 (0.3%)	0 (0%)	2 (0.2%)	1	0.3
Disseminated cancer	8 (1.0%)	5 (4.3%)	13 (1.5%)	0.02	5.41
Open wound/wound infection	1 (0.1%)	1 (0.9%)	2 (0.2%)	0.614	0.25
Steroid use for chronic condition	28 (3.6%)	3 (2.6%)	31 (3.5%)	0.773	0.08
>10% loss of body weight in last 6 months	3 (0.4%)	3 (2.6%)	6 (0.7%)	0.036	4.4
Bleeding disorders	12 (1.5%)	6 (5.2%)	18 (2.0%)	0.025	5.02
SIRS	1 (0.1%)	0 (0%)	1 (0.1%)	1	0.15
Prior pelvic radiotherapy	13 (1.7%)	3 (2.6%)	16 (1.8%)	0.753	0.1
Prior pelvic operations	279 (36.0%)	56 (48.3%)	335 (37.6%)	0.014	6.08

**Table 3:** Preoperative laboratory values Significance defined as p<0.05; p-values were obtained using chi-square and t-test analysis. BUN: blood urea nitrogen; PTT: partial thromboplastin time; PT: prothrombin time; WBC: white blood cell count; INR: international normalized ratio

	No transfusion, n=776	Transfusion, n=116	Total cohort, n=892	P-value	χ² value
Preoperative serum sodium					
<146	772 (99.5%)	116 (100%)	888 (99.6%)	0.976	0.001
≥146	4 (0.5%)	0 (0%)	4 (0.4%)		
Preoperative BUN					
<21	607 (78.2%)	82 (70.7%)	689 (77.2%)	0.092	2.84
≥21	169 (21.8%)	34 (29.3%)	203 (22.8%)		
Preoperative serum creatinine					
<1.6	717 (92.4%)	93 (80.2%)	810 (90.8%)	<0.001	20.28
≥1.6	59 (7.6%)	23 (19.8%)	82 (9.2%)		
Preoperative platelet count					
<149001	776 (100%)	116 (100%)	892 (100%)	-	-
Preoperative PTT					
<36	744 (95.9%)	107 (92.2%)	851 (95.4%)	0.132	2.27
≥36	32 (4.1%)	9 (7.8%)	41 (4.6%)		
Preoperative INR of PT values					
<1.16	749 (96.5%)	108 (93.1%)	857 (96.1%)	0.131	2.28
≥1.16	27 (3.5%)	8 (6.9%)	35 (3.9%)		
Preoperative WBC					
≤11000	776 (100%)	116 (100%)	892 (100%)	-	-
Preoperative hematocrit					
≥36	720 (92.8%)	79 (68.1%)	799 (89.6%)	<0.001	56.66
<36	56 (7.2%)	37 (31.9%)	93 (10.4%)		

The total operation time (p<0.001), length of hospital stay (p=0.024), and days from operation to discharge (p=0.028) were all higher in patients requiring a transfusion (Table 4). These patients were also more likely to have undergone a concomitant procedure (p<0.001), undergone an unplanned reoperation (p<0.001), and been readmitted (p<0.001).

**Table 4:** Operative characteristics and postoperative outcomes Significance defined as p<0.05; p-values were obtained using chi-square and t-test analysis. MAC: monitored anesthesia care; IV: intravenous; ASA: American College of Surgeons

	No transfusion, n=776	Transfusion, n=116	Total cohort, n=892	P-value	t-value or χ² value
Preoperative oral antibiotic prep	10 (1.3%)	3 (2.6%)	13 (1.5%)	0.501	0.45
Concomitant procedure	142 (18.3%)	39 (33.6%)	181 (20.3%)	<0.001	12.58
ASA class					
1	5 (0.6%)	2 (1.7%)	7 (0.8%)	0.506	0.44
2	195 (25.1%)	15 (12.9%)	210 (23.5%)	0.006	7.54
3	533 (68.7%)	87 (75.0%)	620 (69.5%)	0.204	1.61
4	43 (5.5%)	12 (10.3%)	55 (6.2%)	0.072	3.23
Wound classification					
1. Clean	118 (15.2%)	14 (12.1%)	132 (14.8%)	0.455	0.56
2. Clean/contaminated	652 (84.0%)	101 (87.1%)	753 (84.4%)	0.48	0.5
3. Contaminated	5 (0.6%)	1 (0.9%)	6 (0.7%)	1	<0.01
4. Dirty/infected	1 (0.1%)	0 (0%)	1 (0.1%)	1	0.15
Total operation time	176.64 (70.380)	207.99 (95.050)	180.72 (74.739)	<0.001	-3.42
Length of total hospital stay	3.8660 (5.8810)	5.3879 (11.031)	4.0639 (6.7861)	0.024	-1.74
Days from operation to discharge	3.8144 (5.8029)	5.2845 (11.015)	4.0056 (6.7227)	0.028	-1.67
Patient discharged home	746 (96.1%)	106 (91.4%)	852 (95.5%)	0.039	4.26
Principal anesthesia					
General anesthesia	773 (99.6%)	114 (98.3%)	887 (99.4%)	0.257	1.28
MAC/IV sedation anesthesia	0 (0%)	1 (0.9%)	1 (0.1%)	0.271	1.22
Epidural anesthesia	3 (0.4%)	0 (0%)	3 (0.3%)	1	0.45
Still in hospital >30 Days	2 (0.3%)	1 (0.9%)	3 (0.3%)	0.85	0.04
1 unplanned reoperation	17 (2.2%)	15 (12.9%)	32 (3.6%)	<0.001	24.1
2 unplanned reoperations	1 (0.1%)	1 (0.9%)	2 (0.2%)	0.614	0.25
1 readmission	44 (5.7%)	18 (15.5%)	62 (7.0%)	<0.001	13.85
1 unplanned readmission	42 (5.4%)	18 (15.5%)	60 (6.7%)	<0.001	15.21
1 unplanned readmission likely related to the principal procedure	41 (5.3%)	16 (13.8%)	57 (6.4%)	<0.001	11.97
2 readmissions	4 (0.5%)	1 (0.9%)	5 (0.6%)	1	0.15
2 unplanned readmissions	4 (0.5%)	1 (0.9%)	5 (0.6%)	1	0.15
2 unplanned readmissions likely related to the principal procedure	4 (0.5%)	1 (0.9%)	5 (0.6%)	1	0.15

Major complications (p<0.001) and composite complications (p<0.001) were significantly associated with whether a patient required a transfusion. Among preoperative and perioperative complications, chemotherapy within 90 days of the procedure (p<0.001) and the presence of a lymphocele, lymphatic leak, or other fluid collection (p<0.001) were significant predictors of transfusion requirement. Among postoperative complications, urinary leak/fistula (p<0.001), organ/space surgical site infection (SSI) (p=0.003), requiring mechanical ventilation for more than 48 hours (p=0.036), acute renal failure (p=0.019), cardiac arrest requiring CPR (p=0.009), bleeding transfusion (p<0.001), prolonged postoperative nothing by mouth (NPO) or nasogastric tube (NGT) use (p=0.019), sepsis (p=0.009), prolonged length of hospital stay (p<0.001), and prolonged time to discharge (p<0.001) were all significant predictors of transfusion requirement (Table 5).

**Table 5:** Preoperative, perioperative, and postoperative variables Significance was defined as p<0.05; p-values were obtained using chi-square and t-test analyses. SSI: surgical site infection; CVA: cerebrovascular accident; CPR: cardiopulmonary resuscitation; DVT: deep vein thrombosis; NPO: nothing by mouth; NGT: nasogastric tube

	No transfusion, n=776	Transfusion, n=116	Total cohort, n=892	P-value	χ² value
Summary of complications					
Major complications	99 (12.8%)	41 (35.3%)	140 (15.7%)	<0.001	33.49
Minor complications	27 (3.5%)	8 (6.9%)	35 (3.9%)	0.131	2.28
Composite complications	115 (14.8%)	44 (37.9%)	159 (17.8%)	<0.001	35.34
Preoperative and perioperative variables					
Stent placement performed	45 (5.8%)	9 (7.8%)	54 (6.1%)	0.537	0.38
*Clostridioides difficile* occurrence	1 (0.1%)	0 (0%)	1 (0.1%)	1	0.15
Perioperative antibiotic use	765 (98.6%)	115 (99.1%)	880 (98.7%)	0.958	0
Chemotherapy within 90 days	14 (1.8%)	9 (7.8%)	23 (2.6%)	<0.001	14.11
Drain placement performed	568 (73.2%)	90 (77.6%)	658 (73.8%)	0.374	0.79
Lymphocele, lymphatic leak, or other fluid collection	14 (1.8%)	9 (7.8%)	23 (2.6%)	<0.001	14.11
Malignancy present	619 (79.8%)	92 (79.3%)	711 (79.7%)	1	<0.01
Postoperative complications					
Urinary leak/fistula	15 (1.9%)	11 (9.5%)	26 (2.9%)	<0.001	19.56
Ureteral obstruction	1 (0.1%)	1 (0.9%)	2 (0.2%)	0.614	0.25
Rectal injury	2 (0.3%)	0 (0%)	2 (0.2%)	1	0.3
Superficial incisional SSI	12 (1.5%)	4 (3.4%)	16 (1.8%)	0.287	1.13
Deep incisional SSI	2 (0.3%)	0 (0%)	2 (0.2%)	1	0.3
Organ/space SSI	8 (1.0%)	6 (5.2%)	14 (1.6%)	0.003	8.91
Wound disruption	2 (0.3%)	1 (0.9%)	3 (0.3%)	0.85	0.04
Pneumonia	16 (2.1%)	4 (3.4%)	20 (2.2%)	0.545	0.37
Number of unplanned intubation occurrences					
1	3 (0.4%)	3 (2.6%)	6 (0.7%)	0.008	7.02
2	1 (0.1%)	1 (0.9%)	2 (0.2%)		
Pulmonary embolism	6 (0.8%)	2 (1.7%)	8 (0.9%)	0.627	0.24
On ventilator >48 hours	3 (0.4%)	3 (2.6%)	6 (0.7%)	0.036	4.4
Progressive renal insufficiency	16 (2.1%)	1 (0.9%)	17 (1.9%)	0.605	0.27
Acute renal failure	15 (1.9%)	7 (6.0%)	22 (2.5%)	0.019	5.52
Urinary tract infection	15 (1.9%)	4 (3.4%)	19 (2.1%)	0.478	0.5
Stroke/CVA	0 (0%)	1 (0.9%)	1 (0.1%)	0.271	1.22
Cardiac arrest requiring CPR	0 (0%)	2 (1.7%)	2 (0.2%)	0.009	6.82
Myocardial infarction	6 (0.8%)	1 (0.9%)	7 (0.8%)	1	<0.01
Blood transfusion	0 (0%)	116 (100%)	116 (13.0%)	<0.001	892
DVT/thrombophlebitis	7 (0.9%)	1 (0.9%)	8 (0.9%)	1	<0.01
Prolonged postoperative NPO or NGT use	15 (1.9%)	7 (6.0%)	22 (2.5%)	0.019	5.52
Sepsis	4 (0.5%)	4 (3.4%)	8 (0.9%)	0.009	6.82
Septic shock	3 (0.4%)	2 (1.7%)	5 (0.6%)	0.257	1.28
Prolonged operation time	430 (55.4%)	57 (49.1%)	487 (54.6%)	0.244	1.36
Prolonged length of hospital stay	229 (29.5%)	61 (52.6%)	290 (32.5%)	<0.001	22.4
Prolonged days to discharge	222 (28.6%)	59 (50.9%)	281 (31.5%)	<0.001	20.75

In the logistic regression models, patients who received a blood transfusion were more likely to develop composite complications than those who did not (OR: 1.20, 95% CI: 1.01-1.41) (Table 6). Patients who underwent a concomitant procedure had higher odds of receiving a blood transfusion (OR: 2.27, 95% CI: 1.46-3.49, p<0.001). Black or African American participants had significantly higher odds of transfusion (OR: 2.31, 95% CI: 1.10-4.72, p=0.023). Patients with a bleeding disorder (OR: 3.51, 95% CI: 1.17-9.57, p=0.017), a history of prior pelvic operations (OR: 1.66, 95% CI: 1.11-2.48, p=0.014), or a history of chemotherapy within 90 days of the procedure had significantly higher odds of transfusion (OR: 4.89, 95% CI: 1.89-12.22, p=0.001). Those who underwent an unplanned reoperation also had substantially higher odds for transfusion (OR: 6.16, 95% CI: 2.89-13.02, p<0.001) (Table 6).

**Table 6:** Logistic regression model for preoperative, perioperative, and postoperative variables No transfusion was used as the reference. Significance was defined as p<0.05. Controlled variables included age, body mass index, minority status, current smoking status, insulin-dependent diabetes mellitus, history of severe chronic obstructive pulmonary disease, steroid use for a chronic condition, hypertension requiring medication, and bleeding disorders. ASA: American Society of Anesthesiologists; BMI: body mass index; SSI: surgical site infection

	Univariate analysis			Multivariate analysis	
	Odds ratio (95% CI)	P-value		Odds ratio (95% CI)	P-value
Preoperative and perioperative					
Concomitant procedure occurred	2.26 (1.47-3.45)	<0.001		2.27 (1.46-3.49)	<0.001
Dyspnea present	1.97 (0.96-3.74)	0.049		1.83 (0.87-3.61)	0.094
Age of patient					
1^st^ tertile (20-57 yo)	0.64 (0.41-0.98)	0.045		0.72 (0.45-1.12)	0.155
2^nd^ tertile (58-66 yo)	1.03 (0.67-1.57)	0.884		1.05 (0.67-1.61)	0.829
3^rd^ tertile (67-85 yo)	1.47 (0.98-2.18)	0.058		1.3 (0.84-1.98)	0.236
ASA class 3	1.37 (0.88-2.17)	0.17		1.28 (0.81-2.07)	0.300
Black or African American participants	1.67 (0.90-2.92)	0.086		2.31 (1.10-4.72)	0.023
BMI overweight/obese	0.7 (0.41-1.22)	0.185		0.64 (0.37-1.14)	0.115
Bleeding disorder	3.47 (1.19-9.14)	0.015		3.51 (1.17-9.57)	0.017
Prior pelvic radiotherapy	1.56 (0.35-4.92)	0.494		1.46 (0.33-4.73)	0.564
Prior pelvic operations	1.66 (1.12-2.46)	0.011		1.66 (1.11-2.48)	0.014
Insulin-dependent diabetes mellitus	1.61 (0.87-2.82)	0.107		1.31 (0.69-2.36)	0.395
Chemotherapy within 90 days	4.58 (1.87-10.70)	0.001		4.89 (1.89-12.22)	0.001
Postoperative					
Transfusion	1.20 (1.01-1.42)	0.033		1.20 (1.01-1.41)	0.039
Unplanned reoperation	6.63 (3.18-13.71)	<0.001		6.16 (2.89-13.02)	<0.001
Urinary leak/fistula occurred	5.31 (2.32-11.82)	<0.001		5.03 (2.13-11.53)	<0.001
Unplanned intubation occurred	6.89 (1.61-29.52)	0.007		6.94 (1.52-32.73)	0.010
Prolonged operation time	1.29 (0.87-1.90)	0.206		1.3 (0.87-1.95)	0.194
Organ/space SSI occurred	5.24 (1.70-15.34)	0.003		5.58 (1.76-16.80)	0.002
Lymphocele, lymphatic leak, or other fluid collection occurred	4.58 (1.87-10.70)	0.001		5.02 (2.01-12.01)	<0.001
Progressive renal insufficiency occurred	0.41 (0.02-2.05)	0.393		0.45 (0.02-2.34)	0.447
Sepsis occurred	6.89 (1.61-29.52)	0.007		7.72 (1.66-36.16)	0.007
In hospital >30 days	3.37 (0.16-35.40)	0.323		1.89 (0.08-21.81)	0.621
2^nd^ unplanned reoperation	6.74 (0.27-171.21)	0.178		3.63 (0.09-107.35)	0.413

## Discussion

Despite its advantages over radical nephrectomy, perioperative bleeding remains a consequential risk for PN patients [8]. Prior studies cite various reasons for complications related to blood loss and hemorrhage, such as hospital volume, open versus minimally invasive or robotic surgical approaches, tumor diameter, tumor depth, and obesity status [9-13]. Despite these suggested correlations, no prior study has yet completed a thorough analysis to determine the risk factors for receiving a blood transfusion during or after a PN procedure.

Patients with a history of a bleeding disorder, prior pelvic surgery, or chemotherapy within 90 days of surgery were more likely to require transfusion. Patients with bleeding disorders often require platelet or plasma transfusions with clotting factors to prevent anemia from excessive blood loss. It is also known that patients with a history of prior pelvic surgery are more likely to have longer operative times, require reoperation, or experience hemorrhage [14,15]. Each of these factors is more likely to be associated with a patient’s risk of requiring a blood transfusion. Chemotherapy can increase the risk of bleeding as a result of various factors, including bone marrow damage, destruction of red blood cells leading to low red blood cell count, and thrombocytopenia [16]. These findings underscore the importance of considering a patient’s medical and surgical history when assessing transfusion risk. Concomitant procedures were associated with an increased likelihood of transfusion, potentially due to an increased risk of surgical bleeding resulting from ruptured blood vessels or hemorrhage [17]. The link between transfusion risk and concomitant procedures suggests that complex surgical patients with higher morbidity risk may warrant closer monitoring.

In assessing the impact of transfusion itself on the development of composite complications, our analysis indicated that patients who received a blood transfusion had 20% higher odds of experiencing composite complications compared to those who did not receive a transfusion. These findings emphasize that, although potentially life-saving, transfusions are not without formidable risks of their own. Therefore, careful consideration of transfusion practices, such as implementing restrictive transfusion thresholds and exploring alternative strategies, should be an integral part of patient care in PN procedures.

Postoperatively, unplanned reoperations, urinary leaks or fistulas, unplanned intubation, organ/space surgical site infections, lymphocele, lymphatic leaks, or other fluid collections, and sepsis were all strongly associated with an increased risk of transfusion. Patients requiring transfusions may have higher rates of unplanned reoperations due to increased intraoperative blood loss, which can lead to inadequate tissue perfusion, impaired healing, or technical complications that necessitate additional surgeries. Urinary leaks or fistulas may arise more frequently in these patients, as transfusions could reflect significant blood loss or damage to surrounding tissues, complicating urinary tract repair. Unplanned intubation is often a marker of respiratory compromise, which may be exacerbated by transfusion-related fluid shifts, electrolyte imbalances, or prolonged surgeries leading to pulmonary dysfunction. The risk of organ/space surgical site infections may increase, as transfusions can lead to immunosuppression or tissue edema, both of which hinder the body’s ability to fight infections. Lymphocele and lymphatic leaks may occur more often due to intraoperative disruption of lymphatic vessels, with transfusions potentially reflecting a more complex or prolonged procedure, heightening the risk of these complications. Similarly, fluid collections might form due to poor wound healing, which is exacerbated by transfusions, as they are often a marker of significant operative trauma or blood loss. Finally, sepsis is more likely in transfused patients due to a combination of factors, including transfusion-related immunomodulation, which weakens the immune response, as well as the higher incidence of infections in patients with significant operative or postoperative complications. These findings highlight the critical importance of preventing and managing such complications to reduce the need for transfusions and improve patient outcomes.

Strengths of this study include the substantial sample size and standardized data within the ACS NSQIP database. Notably, the database tracks postoperative complications for up to 30 days following surgery, extending beyond most inpatient hospitalizations. However, complications or mortality after 30 days are unknown and thus were excluded from analysis. A major limitation of this study is the unspecified timing of the transfusions. The ACS-NSQIP database lacks precise chronology for transfusion events. Understanding when transfusions occurred would be valuable for a more definitive understanding of whether the variables are predictors or consequences. Additionally, while the database is extensive, it relies on data submitted by participating institutions, making inclusion in the database voluntary. This may result in differences in postoperative outcomes between participating and nonparticipating sites, which impact the generalizability of our results, as crucial representation from more rural or community-based hospitals may not be incorporated. Importantly, the dataset lacks some variables that can explain the risk of transfusion, including tumor complexity and estimated blood loss. The absence of these variables introduces potential residual confounding. Additionally, while preoperative hematocrit differed significantly between groups on univariate analysis, it was excluded from our final multivariable model due to high statistical collinearity with baseline hemoglobin and renal function markers, which represents a limitation.

## Conclusions

Our study is the first of its kind to delve into the factors surrounding transfusion bleeding risk in patients undergoing PN. Our findings underscore the need for a comprehensive risk assessment and careful monitoring of patients undergoing PN. Vigilant monitoring of high-risk factors and proactive management of complications are needed to reduce transfusion-related morbidity and improve patient outcomes. This study found that transfusion following PN was associated with several preoperative and perioperative factors, including bleeding disorders, prior pelvic surgery, recent chemotherapy, concomitant procedures, and unplanned reoperation. Transfusion was also associated with higher postoperative morbidity. Strategies aimed at minimizing complications, such as optimizing patient health preoperatively and ensuring meticulous surgical techniques, may help reduce the need for blood transfusions. However, because of the retrospective design, lack of transfusion timing, and absence of tumor- and surgery-specific variables, these findings should be interpreted as associations rather than causal relationships. Further research and continuous evaluation of transfusion practices are warranted to validate these findings and develop tailored transfusion protocols for patients undergoing PN to enhance overall quality of care.
